# Supramolecular Aggregates: Hardness Plus Softness

**DOI:** 10.3390/molecules26144233

**Published:** 2021-07-12

**Authors:** Lia Queiroz do Amaral

**Affiliations:** Instituto de Física, Universidade de São Paulo, São Paulo CEP 05508-090, Brazil; amaral@if.usp.br

**Keywords:** interdisciplinary perspective, basic concepts, molecular physics, lyotropic liquid crystals, molecular biophysics, biology

## Abstract

The properties of supramolecular aggregates cross several disciplines, embracing the sciences of nature and joining theory, experiment, and application. There are few articles centering on the problems of interdisciplinarity, and this paper gives an alternative approach, starting with scientific divulgation, bringing concepts from their origin, to facilitate the access of young scientists to the scientific content. Didactic examples are taken from the experience of the author in changing directions of research due to several circumstances of life (including maternity), starting from the view of a rigorous student of physics and evolving to several subjects in chemistry. The scientific part starts with concepts related to nuclear interactions, using the technique of neutron scattering in reactors, and evolves to research in molecular physics. Finally, it arrives at the academic context, with research in condensed matter physics, with X-ray and other techniques, starting with detergents forming nematic lyotropic liquid crystals and the phase transition sequence of isotropic to nematics to hexagonal. The scientific subjects evolved to biological and bio-inspired liquid crystals, including DNA and also specific lipids and phospholipids in biomimetic membranes. Special attention is given to the question of distribution of matter in these complex systems and the non-trivial connections between biochemistry, structures, auto-aggregation, and biology.

## 1. Introduction

Hardness and softness are properties of materials on the macroscopic scale but are the result of interactions at the molecular level. Solids are the result of the existence of an atomic structure with atoms fixed at defined positions, while softness results from the fact that atoms have freedom of motion. The degree of freedom and the existence of spatial correlations between different atoms define the differences in macroscopic behavior. For instance, a diamond crystal and the graphite of a pencil are both made of carbon atoms, which exist also as a gas. Water is a liquid existing in the three states of matter by temperature variation, with very complex interactions between only two types of atoms, hydrogen and oxygen. Basic research in nature sciences aims to understand the interactions in matter, while engineering focuses on controlling processes for uses in human activities.

Following the transformations due to the industrial revolution, in the second half of the 18th century, the study of solid materials was introduced formally, by the middle of the 19th century, in engineering courses in Germany and England, and also in the USA. The needs of the nascent industry required knowledge of elasticity and material resistance, together with metallurgy and crystallography. Focus was also given to changes in the material properties with changes in the macroscopic variables of a system, for example, changes from a solid to a liquid, from a liquid to a gas, or between different phases of metals and alloys. Basic books on these fundamental issues are necessary for graduate students to enter these fields, especially from a theoretical point of view [1,2]. Note that [1] has the view of metallurgy (not the exactness of crystallography) while [2] is for a general audience, and none of them are “for crystallographers”, and that is a first challenge towards multidisciplinary issues.

Scientific studies of supramolecular aggregates evolved initially in two different directions: colloid science and supramolecular chemistry, both starting before the turn of the 20th century. In the last decades, this interdisciplinary field, with the study of intermolecular bonding, has included systems that self-assemble, at the triple meeting point of chemistry, biology, and physics [3], and has reached the 21st century with an incredible power in terms of both basic knowledge and technological applications. It is indeed a long history, connected to deep transformations in human societies!

There are many specialized articles on specific aspects of subjects in the frontiers of science and their technological applications. However, there are few articles centering on the problems of interdisciplinarity, with a comprehensive approach for students and beginners. Knowledge evolves in each discipline starting from its specific criteria, and there are no conceptual bridges among them, so conditions for synthesis are not trivial.

The approach between neighboring research fields is in general obtained from methodologies and techniques which can be used in both fields, but this, in general, means that close disciplines may diverge, even when using the same techniques. The best example is the parallel existence of physical chemistry and chemical physics, with an uneasy dialogue between them. The problem with biology is much bigger, since there is not even a basic conceptual starting point in common. 

This paper aims to offer an alternative approach to this difficult problem, focusing on basic concepts necessary to enter interdisciplinary fields, with an initial approach of scientific divulgation. 

Studies of colloids started in the early 1800s. The word is derived from the Greek “kola” (glue) and was used to distinguish colloidal solutions from solutions with crystallized particles (such as sugar and salt). A colloidal solution is a heterogeneous system consisting of a mechanical mixture of particles with sizes between 1 and 1000 nm, in a continuum medium (solid, liquid, or gas), with behavior that is intermediate between a true solution and particles dispersed in a medium (suspension, emulsion, or foam). This interdisciplinary field evolved to studies of the reactivity of surfaces and interfaces, making a bridge between the macroscopic level and the atomic and molecular levels. It can be said that colloid and interface science dealt with nanoscale objects for nearly a century before the term *nanotechnology* was coined [4]. Interface and colloid science have applications and ramifications in the chemical industry, pharmaceuticals, biotechnology, ceramics, minerals, nanotechnology, and so on [5].

Beginning in the 19th century, liquid crystals (LC) also have a long history. In 2013, their 125th anniversary was celebrated [6]. This article of fewer than five pages gives 10 basic references to those wishing to enter the field, providing the foundations on thermotropics (temperature-dependent), lyotropics (composition-dependent), and applications (from displays to biology).

Supramolecular chemistry can be considered to begin in the late 1800s when the German organic chemist Hermann Emil Fisher, studying purines and sugar, suggested that enzyme/substrate interactions take the form of a “lock and key”. He received the Nobel Prize in 1902. The fundamental principles of molecular recognition and host–guest chemistry developed, and in the early 20th century, intermolecular bonds started to be understood in gradually more detail, through a merging process with quantum physics, a difficult process [7].

In the early 1900s, physics found its way focusing on quantum mechanics and turning to the microscopic world below the level of the hydrogen atom, while chemistry focused on molecules and above, turning towards the human world, and chemical industries helped to change human societies. Quantum chemistry dominated the scene in chemistry until the discovery of the structure of DNA in the 1950s. The second half of the 1900s saw the emergence of biochemistry and molecular biology—a serious attempt at integration of the natural sciences, posing an enormous challenge for graduate education [8]. 

Let us now turn back to some specific supramolecular aggregates: polymers and macromolecules, which are not the same thing. The word “polymer” (from the Greek polys meaning “many” and meros meaning “part”) was first introduced in 1833 by the Swedish chemist Jöns Jakob Berzelius, while the German chemist Hermann Staudinger felt it necessary to coin the word “macromolecule” in 1922 to describe large covalently bonded organic chain molecules containing more than 103 atoms [9].

Since pre-history, human activities have involved the use of materials, both natural and modified, many of them containing polymers (such as wool, cotton, fibers, etc.). Chemical manipulation of such raw materials started in the 19th century, without knowledge of their nature. Initial rationalization was proposed by the Scottish chemist Thomas Graham (Royal Society medals in 1838 and 1850), considering them as colloidal aggregates of small molecules, held together by unknown forces. Even without theories, the potential of polymers in industry was grasped and used. The German chemist Hermann Staudinger proposed in 1920 that polymers were in fact long chains of atoms linked by covalent bonds, receiving the Nobel Prize in 1953. The subsequent discovery of biopolymers, natural polymers produced by the cells of living organisms, opened the Pandora’s box of biochemistry and molecular biology. 

Main-chain and side-chain polymers can be both lyotropic and thermotropic LC polymers. The chemical structure of a polymer backbone, either as a single polymer or in bulk, is often not suitable to be used in practical applications. Polymer–dye conjugations are one of the common examples of polymer modifications. They represent a crucial step for imaging in optical microscopy or for tracing and marking macromolecules. A comparison of recent original strategies to conjugate polymers to ligands and dyes (e.g., for detection or targeting purposes in health care applications) can be found in [10].

Up to here, this survey has given just a snapshot of the problems faced nowadays by young scientists at the crossing of the so-called sciences of nature. This review turns now to my own trajectory, as an example of the difficulties faced in the transition from the student level to the professional level in science, particularly for a woman, in a peripheral country, entering middle school around 1955 and good in mathematics. 

My trajectory touched eventually on polymers, but that was not my expertise. I have worked mainly at the interfaces of physics/chemistry/biology/education, and at the interface of natural sciences/human issues. I do not mention all the research developed along my career, but several of my own papers are given as references, when having direct relation to the subjects discussed. 

As a student of physics, I started a successful initiation of research in nuclear physics, and immediately after graduation I secured a job working in a research reactor, related to the technique of slow neutron scattering, where I stayed for 12 years. However, I quit that job after my daughter was born. Later on, it was possible for me to restart from zero, with a provisional part-time job at the Institute of Physics of USP (IFUSP), working in other directions. Only several years later was I able to return to the data from the “neutron time”, when I was again in full-time research in condensed matter physics, with tenure at IFUSP.

This review emphasizes interdisciplinary concepts from the beginning of my career, which are good for didactical explanations of the changes of direction I needed to face. This is included in Section 2, along with some more personal issues, while Section 3 deals with more objective scientific discussions on obtained results from the neutron time. Section 4 deals with the project I started in IFUSP, creating a new X-ray Laboratory of Crystallography and initiating research on lyotropic liquid crystals, while Section 5 takes the discussion in the direction of biology, focusing more on the present. 

## 2. Theory vs. Experiment

The discussion of the relative importance of theory and experiment has no trivial answer; knowledge is constructed from both. Sometimes an experiment is planned to test a theory, but many times an experimental result is obtained for practical reasons, and the search for a theoretical explanation comes later on. This depends also on the field being considered, and on its historical development.

It should be stressed that what are called “exact sciences” are the result of the union of physics with chemistry at the turn of the 20th century. Modern physics came after the definition of chemistry as a true science, with the construction of the periodic table of elements. Physical theories are more focused on mathematical rigor and “elegance”, while chemical theories are usually more connected to experimental results. 

Around the year 1940, areas of interest to physics and engineering were grouped together as solid state physics, with the development of semiconductors leading to micro-electronics. Materials science started to become an extremely vast multidisciplinary field, defined by the triangle structure–processing–properties, with applications in engineering and industry. 

Condensed matter physics started around 1960, when the study of liquids started to become relevant. Simple liquids, made of a single component, started to be investigated by physics, but solutions with multiple components were subjects of physical chemistry. 

This review will now consider the perspective of a young female (YF) student of physics, very rigorous in mathematics, initiating scientific research in 1960, in the middle of ideological conflicts with external reality. This section will chronologically follow the YF’s experiences (***important points stressed in bold italics***).

### 2.1. In Nuclear Physics

YF started to do research in a large project on monazite sands from Brazil, connected to the local chemistry industry, with the specific aim to study, by slow neutron transmission, the total cross-sections of some rare earths of interest to nuclear energy in connection with the production of thorium [11]. The experimental steps in the nuclear research reactor included getting the samples; conditioning them in sample holders; making all measurements at the reactor; understanding how the neutron beam was obtained and how to change its energy; using a crystal monochromator, and a neutron mechanical velocity selector, both locally constructed; making transmission measurements with the detector; and using the available electronics. Of particular interest was the resonance curve of lutetium (Lu) as function of neutron energy. In parallel, YF attended classes on nuclear physics and quantum mechanics (QM), and also the first course on computers given by IBM in Brazil, learning to program in Fortran and using an IBM 1620 computer for data analysis.

Then, however, YF realized that the theoretical courses did not directly help the analyses, as there was no clear route to join theory with obtained data. It was clear that Breit–Wigner theory [12] should be used to interpret the experimental results for the resonance in Lu. However, QM scattering theory had possible solutions only in the first Born approximation, which was not valid for slow neutrons. The problem seemed untreatable since the true nuclear potential was also unknown. YF found out that a genial solution is the pseudo-potential introduced by Fermi [13], which solves the untreatable problem of scattering of slow neutrons by using a delta function and the known experimental result for the scattering length. With such a strategy, the project arrived at a result useful to nuclear energy, presented by YF at an international local congress promoted by the Brazilian Atomic Energy Committee together with the International Atomic Energy Agency (IAEA, 1963). Several years later, it resulted in an academic publication [14]. 

***The solution is to escape from an untreatable, exact theory by using a correct conceptual solution together with an experimental result.***

### 2.2. In Molecular Physics

YF, after research on nuclear physics with slow neutrons in Brazil, was sent on a fellowship with IAEA to Sweden, alone in 1964, to work in the small group of Prof. Karl-Erik Larsson at the Royal Institute of Technology, Department of Reactor Physics, in Stockholm, and stayed there for 15 months. The samples were hydrogenous liquids, studied by cold neutron incoherent scattering, and the experimental setup included a filter of beryllium refrigerated by liquid nitrogen inside the reactor beam, used to extract the cold neutrons, while a chopper with time-of-flight made the energy analysis. Not in a transmission geometry but with angular variation in order to also measure momentum transfer.

***YF’s personal life was very difficult in Sweden, an experience of hard loneliness, but also of freedom and independence.***

After some time reading the literature, YF could grasp the theoretical problem. It was the QM theory for correlations in space and time and Born approximation scattering in systems of interacting particles, the Van Hove formalism [15], using the Fermi pseudo-potential for the neutron–nucleus interaction. However, this was extended to all neutron energies by describing the Born approximation scattering in terms of the time-dependent pair-distribution function G (r,t), a very natural extension of the conventional static g(r) function. 

***Exact, beautiful, and understandable, but to apply that theory to complex hydrogenous molecular systems was again an intractable problem!***

Prof. Larsson was working on a theoretical model for the derivation of a neutron-scattering cross-section for quasi-elastic scattering from a complex hydrogenous liquid, separating the proton motion within the molecule from the motion of protons between molecules. The foreign students, however, worked on data from specific samples. The Be filter produced an incident beam with a sharp edge, due to a Brag peak [16] and a continuum cold spectrum for higher wavelengths. Analyzed samples showed a broadening of the sharp edge, related to a quasi-elastic scattering. Hydrogenous liquids also presented an inelastic part, which was not focused by the group.

The work in Sweden was published later on in Physical Review. It included a first section by Larsson with Bergsted, on theory [17], and a second section by Larsson and the foreign students, on experimental results related to pentane and *n*-propyl alcohol [18].

***Only after seeing the final paper, one year later, could YF really understand what was done by the whole group, and her role in all of it.***

### 2.3. In Getting Academic Degrees

Back home, YF focused on transforming the previous experimental results on rare earths into an academic paper [14]. 

At the same time, results started to be obtained with the new equipment, which was similar to the Swedish equipment, focusing first on the calibration and resolution of the chopper time-of-flight spectrometer, with a detailed mathematical analysis of the problem. These results were published as a paper [19]. Neutron data was also obtained in solid materials (uranium oxide and polycrystalline iron) and in a large series of hydrogenous liquids. Several papers of the reactor group were presented in the first meeting of the Brazilian Physical Society (1966).

In 1968, a big change in internal politics occurred in academic life in Brazil. The European system, based on academic chairs with professors and their assistants, was being replaced by the American system, with post-graduate courses, requiring qualifying examinations and credits before Master and Doctorate degrees. At the same time, all the departments of physics existing in faculties had to move towards independent institutes of physics.

YF took the opportunity to get a master’s degree in Nuclear Science and Engineering, wrote a dissertation based on the published paper [19], presented and defended it in the new American system (June 1969), and was taken on to complete her Ph.D. in Physics, still in the European system, with a rigid deadline: the end of 1972.

For the thesis, YF found a suitable compound in a handbook of chemical products: a plastic crystal with phase transitions near room temperature. Data was obtained through two years of experimental work. Already pregnant, YF worked hard to write the thesis, and gave it the flavor of molecular physics, also analyzing inelastic neutron differential scattering.

The Ph.D. thesis was defended at the end of November 1972, and maternity occurred five days later. Since it was impossible to reconcile her job hours with her personal life, YF decided to quit the nuclear reactor to stay at home.

### 2.4. In Changing Directions

The change in direction meant a definite change in technique since slow neutrons were available only at the reactor. The opportunity to start a part-time job at IFUSP meant giving classes on basic physics (theory and laboratories) for the initial two years of the engineering courses. This also entailed a proposal for research, starting a new X-ray crystallography laboratory (CrysLab) together with a colleague that had just finished a Ph.D. focused on defects in crystals.

The basic theory for the interaction with matter is rather different. Slow neutrons interact with the nuclei treated with the Fermi pseudo-potential, while X-rays interact with the electrons via classical electromagnetism. Furthermore, slow neutrons interact mostly with hydrogen, via incoherent scattering, while X-rays interact with crystals via coherent scattering. The previous expertise of YF with neutrons was of little help.

For the Ph.D. thesis, YF had some interaction with the chemistry department of USP, since in physics, the focus was on the theoretical QM of the H atom, not of molecules. Looking for a new subject of research, YF again went there, and, by chance, attended the seminar of a Canadian chemist who was visiting the NMR group and who worked with lyotropic nematic liquid crystals.

From this beginning, added to the previous experience with hydrogenous liquids and plastic crystals, YF was able to define a research project on lyotropic liquid crystals (in close connection with bio-membranes). It would be a collaboration with the NMR group of the Chemistry Institute, where the samples (aqueous mixtures of water/detergent/additives) were prepared. The NMR research was focused on the structure of molecules (such as benzene) oriented by the lyotropic systems, while the IFUSP project proposed X-ray structural study of the lyotropic phases. 

The whole project had no connection with the research done at IFUSP. It was presented to a financial agency within a general proposal to open IFUSP to new areas of research. It took two years for the equipment to arrive, and during this time, YF returned to the neutron data, in order to develop scientific papers. The result is objectively presented in the next section of this review.

## 3. Conformations of Hydrocarbon Chains by Slow Neutron Scattering

This subject was chosen to receive special attention in this review since it provides an opportunity to discuss objective results obtained with the technique of neutron scattering in the years’ 1960s.

Studies of rotational and vibration degrees of freedom by molecules using spectroscopic methods were already well established by the year 1960 [20]. However, for those working with hydrogenous materials with slow neutrons using research reactors, an alternative approach became available through analysis of the total neutron scattering cross section per H atoms, σ_s_/H, with a defined slope as a function of neutron energy [21]. The model, based on the Fermi pseudo-potential, describes the scattering in terms of two molecular parameters: an effective proton mass for translation and rotation, and a vibration constant equal to the mean square zero-point vibration displacement of the proton, with good results in the cases of CH_4_ and H_2_. Measurements of ammonium halides have also shown a definite correlation between the slope of the cross-section of low-energy neutrons and the rotational freedom of the ammonium ions NH_4_ [22].

### 3.1. In Hydrogenous Materials in São Paulo

The neutron transmission methodology has been used in the study of rotational freedom of a large series of hydrogenous liquids in the research reactor in São Paulo [23]. 

The total cross-section σ = *ln* (*T*^−1^)/*n*, where *T* is the measured neutron transmission and *n* is the number of molecules/cm^2^, is mostly incoherent. The scattering cross-section per H atom σ_s_/H is obtained by correcting for absorption and dividing by the number of H atoms in the molecule. The neutron transmission methodology is based on a calibration curve correlating the slope of σ_s_/H to the activation energy of the rotation being considered for a series of molecules, using the Krieger–Nelkin (KN) model [21], eventually with improvements. The slopes of σ_s_/H, in fact, are not directly connected to the barriers for rotation, but rather related to the energy states below the barrier available for energy-gain scattering by the cold neutrons.

The alcohol molecules in the liquid phase are connected via hydrogen bonding, and differences in the measured slopes reveal free rotation and hindered rotations in CH_3_ and CH_2_ groups [24]. In the cases of methanol, at room temperature, the neutron quasi-elastic scattering results give relaxation times, and the activation energy is in agreement with results from microwave methods [25]. 

### 3.2. In the Polymer PDMS

**Figure 1:**
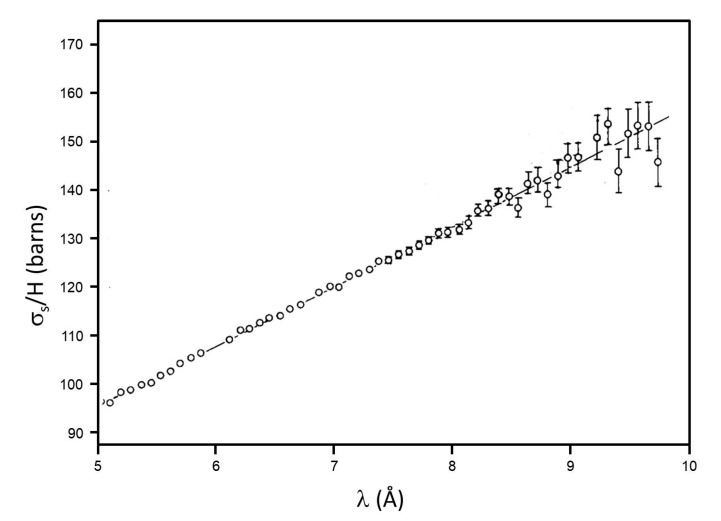
Total scattering cross-section per H atom, σ_s_/H, at room temperature, for PDMS (polymer polydimethylsiloxane) as a function of the neutron wavelength λ. Experimental points and adjusted straight line, with slope related to the barrier for CH_3_ rotations.

With the simple neutron transmission methodology, a study of the CH_3_ rotations in the polymer polydimethylsiloxane (PDMS) at room temperature has been also performed at the São Paulo reactor, and is discussed here in some detail. This silicone, (C_2_H_6_OSi)_n_, has linear units (CH_3_)_2_SiO and terminal groups (CH_3_)_3_SiO. The almost free rotation of the CH_3_ groups was already accepted, but evidence was found also for chain rotation and self-diffusion. NMR results could not exclude hindered rotations of CH_3_ with a small barrier. Slow neutron transmission, a very simple measurement, could, however, give some answers to the problem. This work aimed to clarify different interpretations of the inelastic neutron spectra. In the published paper [26] a figure shows the curve relating the slopes to the KN mass parameter. Here the measured σ_s_/H for PDMS as a function of the neutron wavelength λ is given in Figure 1, showing a very good linearity over the range 5–10 Å, with a slope 12.2 ± 0.2 barns/Å. 

Known calibration curves [22,23,26] give a limit of 0.4 kcal/mole for the barrier hindering methyl rotation in PDMS, since corrections for other low-frequency motions of the molecules in the liquid state would result in a small reduction in the slopes attributed to internal rotation, but these corrections are difficult to estimate. The paper [26] includes some considerations on other contributions, due to larger rotational mass. The conclusion is indication of practically free rotation of CH_3_ groups about their C_3_ symmetry axes. Such unusual freedom was attributed to the greater radius of the silicon atom compared to the C atom, so that the Si-C bond is longer than the C-C bond, and also to the separation between methyl groups, due to the presence of oxygen atoms along the chain skeleton.

It is interesting to mention that this simple transmission result was cited afterwards in a paper comparing the techniques of neutron inelastic scattering and NMR for PDMS below room temperature [27]. Satisfactory agreement of the two experimental techniques is achieved as regards the activation energy and the pre-exponential factor of the Arrhenius approach for the correlation time and jump time. It can be added that PDMS is nowadays used for such large and numerous applications that it is no longer restricted to academic research, and has become the target of industrial design [28].

### 3.3. In a Plastic Crystal

It is convenient to turn now to the study of a molecule classified as a “globular compound”, with a plastic phase intermediary between solid and liquid, where rotations of CH_3_ are expected to be free: tert-butanol (C_4_H_9_OH) or (CH_3_)_3_-C-OH. The study of its molecular dynamics by slow neutron scattering was the object of a Ph.D. thesis in the research reactor in São Paulo [29], with two articles published afterwards [30,31]. A combination of measurements of the total cross-section, by transmission, and of the double differential scattering cross-section as a function of changes in both momentum and energy transfer, was performed with temperature variation. Neutron transmission results were obtained in the temperature interval 0–40 °C, where the phase transitions occur [30], and later, the quasi-elastic and the inelastic scattering provided more information on the molecular dynamics [31].

Abrupt changes in σ_s_/H as a function of temperature may be attributed to the inelastic component and give information on changes in the freedom of motion of molecules and molecular groups at state and phase transitions. Slow neutrons have a short interaction time (ca. 10^−12^ s) and are especially valuable for studying CH_3_ rotations because of the high hydrogen scattering cross-section and large amplitude of motion of the protons. In the case of hindered rotations, the CH_3_ fundamental torsional frequency is easily seen in inelastic neutron scattering spectroscopy, while it is very difficult to detect in infrared and Raman spectra. In view of such characteristics, the molecular dynamics of tert-butanol in two crystalline phases (with a transition at 13 °C), and in the liquid state (melting transition at 23 °C), were investigated by cold neutron scattering. 

Here, the transmission results are discussed in some more detail. The article [30] shows only the transmission measurements versus temperature as averages over 22 series of cooling and/or heating. Results indicate an abrupt step in cooling and a softer one in heating at the change of state, as well as indication of a third crystalline form. Here, the focus is on the barrier to the rotation of the CH_3_ groups, which requires consideration of the bulk density of the sample, with discontinuities at the state and phase transitions, as shown in Table I in [30]. Since it was not possible to know if differences in the sample thickness occurred at the state transition, the two limiting values were considered in the solid state. 

**Figure 2:**
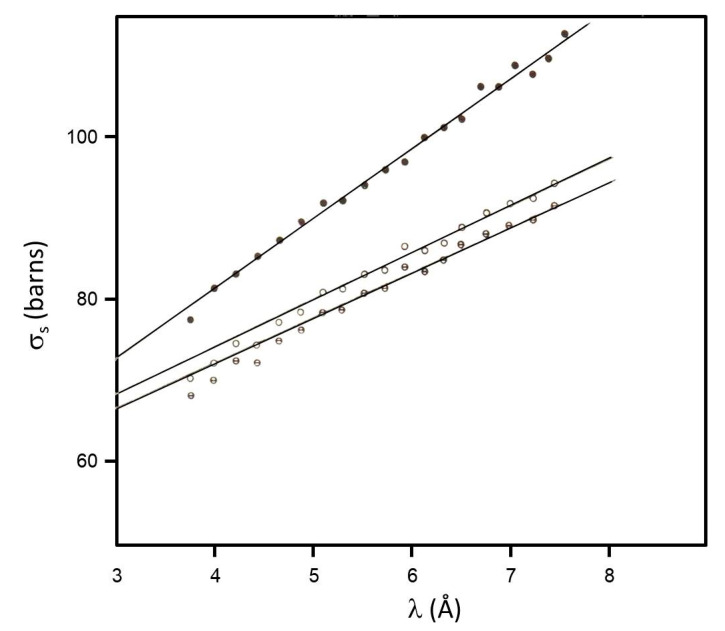
Total scattering cross-section per H atom, σ_s_/H, for tert-butanol, as a function of the neutron wavelength λ. Results for the liquid (upper) and for the two limiting values of the solid state. Straight line adjustment is well defined over the whole λ interval in the liquid state, and for λ > 5 Å in the solid state.

Figure 2 shows the results for σ_s_/H as a function of neutron wavelength λ, for the liquid state and for the two limiting values of the solid state. Straight line adjustment is well defined over the whole λ interval in the liquid state, giving a slope of 8.6 ± 0.2 barns/Å. In the solid state, the two limiting values are very near, the average value was considered, and the adjustment for λ > 5 Å gives a slope of 5.7 ± 0.3 barns/Å. The passage from the slope to the barrier V was made according to two different calibration curves: one due to rotation of NH_4_ in the solid state [22], and the other due to rotation of CH_3_ in associated liquids [23]. The different values correspond to the existence of whole molecule movements in the liquid phase, and the conclusion is that, for tert-butanol, the calibration curve given in [22] should be used for the solid phase, giving V = 3.8 ± 0.5 kcal/mol, while the calibration curve given in [23] should be used in the liquid phase. The overall conclusion from the transmission measurements is that the internal rotation of the methyl group is not sensible for the state and phase transitions, being practically independent from intermolecular forces. 

The more detailed study of the inelastic cold neutron scattering (INS) technique is useful, especially as a complementary technique to infrared or Raman spectroscopy. Oscillations due to internal rotations are, in general, not observed in spectroscopy, since they induce small variations in dipole moment, besides having low frequencies, usually in the microwave region, and being accessible in a gaseous state. By INS, it is possible to study both the energy levels inside the potential well and the relaxation processes from jumps and molecular reorientations. In the results obtained with INS in the study of tert-butanol [31], a frequency spectrum as a sum of seven Gaussian functions fitted the measured time-of-flight distribution and allowed assignment of peak positions. A barrier V = 4.0 ± 0.2 kcal/mol for CH_3_ internal rotation was obtained, in agreement with the result from transmission, within the range of statistical errors. Quasi-elastic line broadening and Debye–Waller factors were analyzed in terms of models for molecular diffusion, and the results were compared with NMR data. It was concluded that cooperative rotational diffusion occurs in both solid and liquid states.

## 4. Self-Assembly and Lyotropic Liquid Crystals

Research on lyotropic complex systems (water/amphiphile/additives) requires a very large amount of previous knowledge of physics and chemistry. Focus on experimental concepts is necessary, besides some knowledge on theories. A purely theoretical approach is still unable to deal with the very complex multicomponent aqueous solutions to be discussed now.

Starting from physics, a good background in the states of matter and their phase transitions is required—of concepts of order and disorder in structures of crystals and polycrystalline, and of phase diagrams as a function not only of pressure and temperature (as for a single component) but also of relative concentration in multi-component systems, as in metallurgy. 

Starting from chemistry, the focus must be on water and its anomalies, the structure of the water molecule and the effects of the H bond, ionization of water and pH, hydrophobic and hydrophilic effects, formation of micelle aggregates by self-assembly, critical micelle concentration, behavior of soaps and detergents in water solution, and phase diagrams as a function of relative concentration and temperature.

**Figure 3:**
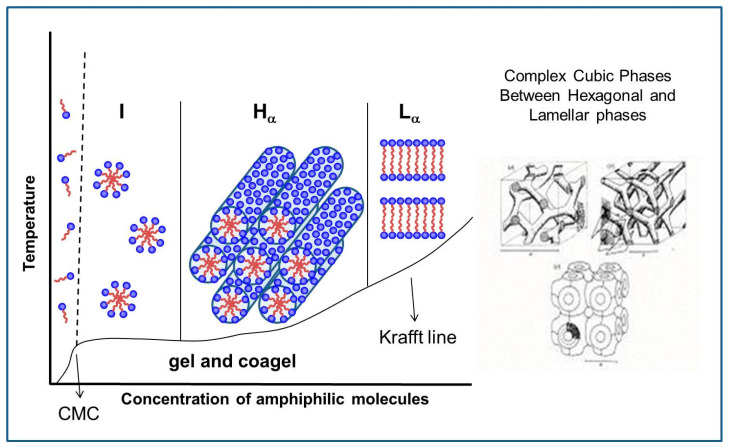
Scheme of phase diagram as a function of concentration and temperature for a lyotropic aqueous system. At the CMC, single molecules form spherical micelles in the isotropic I phase; with increasing concentration, hexagonal Hα and lamellar Lα phases may form at temperatures above the Krafft line that separates these structures from the gel and coagel crystalline phases. Complex cubic structures may form between the H_α_ and L_α_ phases.

Figure 3 provides some idea about micelles, the transient aggregates formed in equilibrium with monomers in solution, changing forms with concentration and temperature. At the CMC, single molecules form spherical micelles in the isotropic I phase; with increasing concentration, hexagonal H_α_ and lamellar L_α_ phases may form at temperatures above the Krafft line that separates these structures from the gel and coagel crystalline phases. Complex cubic structures may form between the H_α_ and L_α_ phases.

An important point to emphasize is that the hydrocarbon chains (HC) inside the aggregates in lyotropic liquid crystals (LLC) are in a disordered α state above the Krafft temperature, while they are in extended β conformation below that temperature. The state of the HC chains can be determined from X-ray diffraction, from the peak corresponding to the average distance between the HC chains: diffuse at 4.5 Å in α state and sharp at 4.1 Å in β state.

### 4.1. The Beginning of the CrysLab IFUSP

Due to the lack of technicians in the group, it was necessary to “became an experimentalist” in order to assemble the electro-hydraulic infrastructure of the lab with diffractometers. It was also necessary to understand the basic physics of the interaction of X-rays with matter.

The technique of X-rays used in the CrysLab corresponded to the usual elastic coherent scattering, where the incident low-energy X-ray photon with wavelength λ induces electron vibration in the same frequency, and the vibrating charged electron emits a photon, in a scattering angle 2θ in relation to the incident photon. The momentum transfer is measured in terms of the scattering vector modulus q = 4π sin(θ)/λ, and the intensity is recorded by photographic technique or by electronic detector. The scattering vector has a defined direction in space. The interaction occurs with the region of the sample illuminated by the X-ray beam, and during the whole exposition time. 

The measured intensity can be written as S(q) = P(q) × S(q), where P(q) is the form factor of the individual scatter, and S(q) is the structure factor of the scatter ensemble. These functions may be continuous or present peaks, corresponding to distances *d* = 2π/q. The term “X-ray diffraction” is used when the sample is a crystal with a well-defined structure and oriented Brag peaks related to the translational symmetry. When the individual scatter is a “particle”, S(q) refers to the ensemble of particles. The term “small angle X-ray scattering” (SAXS) is used when the units are large enough to require small 2θ values. In view of the size of the lyotropic structures, the CrysLab project required both SAXS and diffraction. 

For liquid crystals, the situation requires careful analysis of X-ray results, since the scattering shows, in general, few peaks that may depend on the sample orientation, observed by polarized optical microscopy (POM). 

The first paper of the X-ray CrysLab of IFUSP was in the field of LLC, and was directly connected to the seminal work by the Canadian chemist who found the two uniaxial nematic lyotropic phases [32]. It was with a phase Nd of sodium decyl sulfate, and it was also the first international article on the structure of a nematic LLC [33]. The initial samples (and publications) were made using the recipe of the chemists, adding decanol and/or salt to only two detergents, which were difficult to synthesize or buy (sodium decyl sulfate and potassium laurate), and diffraction was detected by bidimensional film images.

### 4.2. Introduction to Physical Chemistry

Working full time at IFUSP after tenure was hard. It was difficult to reconcile my personal life with teaching and the academic bureaucracy, in addition to research in the CrysLab. My introduction to nematic LLC was due to a “chance event”. I was not, in fact, fully prepared for the difficulties of the field. However, I did my best to fulfill the tasks. Articles were published, one master’s degree was finished, one Ph.D. in LLC was completed, and I started to work hard to get the Dozent frei (DF) academic degree (as exists in Germany), mandatory for a scientific career at IFUSP.

When a biaxial nematic lyotropic phase was discovered experimentally in the USA [34], several groups of physicists entered the field. However, the ternary system is too complex for physicists, and it became clear that the available theories were unable to explain the biaxial phase that is intermediate between the Nc uniaxial phases (cylindrical micelles) and the Nd uniaxial phases (discotic micelles). 

I then realized that I really needed to engage with physical chemistry by myself in order to proceed in LLC. I then studied the DLVO theory on forces between charged surfaces interacting through a liquid medium. I focused on the planar geometry with results in the Nd phase—passing the Nc phase (with potassium laurate) to the Ph.D. student—and the work on interactions between micelles, considering Van der Waals attraction, Coulomb repulsion, and excluded volume gave interesting results [35]. A following master’s degree studied the interaction between surface and magnetic orientation [36].

For my DF thesis, I worked alone to understand the process of magnetic orientation of the micelles due to the HC chains [37], the structure of the gel phase with Bragg peaks at lower temperature [38], and how to use other techniques (thermal analysis and electron microscopy) in LLC [39].

Over two decades (graduation in 1962, Ph.D in 1972, DF in 1982), the student with mathematical rigor turned into a “multitasks” scientist at the interface of physics/chemistry.

This first decade of the CrysLab ended in 1985, when I became Associate Professor at IFUSP, after participating in several international congresses. At that time, the CrysLab had already several published papers and formed two master’s and two Ph.D. students in LLC. The last student focused on elastic constants, obtained from measurements of the critical magnetic field for the Freederickzs transition. It became clear, however, that the lab needed to be renewed, the old hand-control required automation, and a broader direction of research needed to be defined.

### 4.3. Micelles and Interface Curvature

A parallel didactic explanation is now necessary. “Amphiphilic” refers to a molecule made of two distinct covalently bonded components with different affinities for the solvent. One part possesses a high affinity for polar solvents (such as water), and another part has a strong affinity for nonpolar solvents (such as hydrocarbons). Examples of amphiphiles are molecules with hydrophilic and hydrophobic parts, with a paraffin tail and a polar head, such as detergents and some lipids. Grease or oil are not dissolved by water due to their opposite polarity, but are dissolved by aqueous solutions of amphiphiles. A micelle is an aggregate of amphiphile molecules formed spontaneously at a critical micellar concentration (CMC), observed experimentally by a drop in the superficial tension at the interface between the aqueous solution of amphiphiles and air. Micelles are formed inside the solution when monomers fill the interface with air, and are in chemical equilibrium with monomers inside the solution. Surfactants (surface active agents), known as soaps, are amphiphiles that have been used for cleaning purposes since antiquity.

It is possible now to enter in some aspects of interface curvature. The changes in the form of micellar structures require new concepts, and these came in 1976 through the concept of the molecular packing parameter, associating the molecular geometry with the polar–apolar interface curvature of the micellar aggregates. Defining **V** and **ℓ** as the volume and the length of the paraffin tail and **a** as the surface area per molecule, at the interface, the “natural” parameter is defined by **po = V/a ℓ**.

The geometric definition gives a rough idea of the expected curvature of the surfactant aggregate (<1/3 for sphere, 1 for planar bilayer, >1 for inverted micelles). It must, however, be stressed that this parameter by itself does not solve the problem of the form of micellar aggregates, since other factors, such as amount of water bound to the polar head, may drastically change its actual value in a given aggregate. It is out of the scope of this review to enter into the problem of curvature free energy, a problem solved for membranes in vesicle geometry, considering bending rigidity and Gaussian curvature.

However, something about this issue is mentioned in Section 4.5.

### 4.4. Broader Research Directions of the CrysLab IFUSP

Detailed characterization of nematic phases with both optical and X-ray techniques were developed, and automation of the electronics of all the equipment, required much focus on the experimental aspects of the research. At the same time, the research lines opened in the 1990s in the more general direction of “non-crystalline materials”, and new techniques were used, together with new collaborations, also at the international level. 

**Figure 4:**
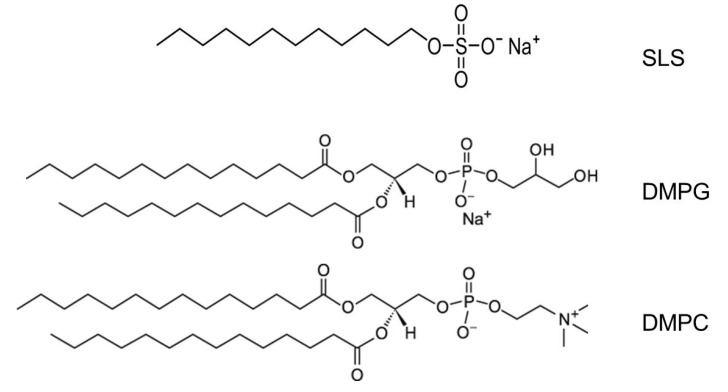
Molecules studied: detergent SLS (sodium dodecyl sulphate) with one HC chain, and phospholipids with two HC chains, charged DMPG and neutral DMPC (item 5.1).

Regarding LLC, it became clear that it was necessary to discover the lyotropic nematic domain in another system of broader interest, and the choice was to focus on sodium dodecyl sulfate (SDS), also named sodium lauryl sulfate (SLS), chemical formula NaC_12_H_25_SO_4_, the standard amphiphile used for micellar systems in physical chemistry, which is easy to buy. It is a simple hydrocarbon chain (*n* = 12), with a terminal CH_3_ and a polar head, as displayed in Figure 4.

With some effort, using POM and surface effects in capillaries, it was possible to discover (by trial and error) an N_C_ phase at 23 °C with weight composition 25.00% SLS/70.53% H_2_O/4.47% decanol [40]. A systematic search of the nematic domain with SLS was then made as a function of two concentration variables, the water/amphiphile (Mw) and the decanol/amphiphile (Md) relative molar ratios, with good results [41]. The ternary phase diagram at room temperature showed the nematic domain between the isotropic I, hexagonal H_α_, and lamellar L_α_ phases already studied by chemists. The phase diagram as a function of temperature and Md indicated a change in micellar form at the transition Nd–Nc, for Md = 0.38, with Mw varying in the interval 40–45. Furthermore, a comparison with sodium decyl sulfate and K laurate in terms of the new variables Mw and Md (Table II of [41]) revealed that the transition between the two uniaxial forms occurred for very near Md values, while Mw had a large variation (from 21 to 43), depending on the size of the micellar aggregate. These original results defined new directions for the research.

The diversification of directions of research included a master’s degree on the isotropic phase of the binary system SLS/water, by SAXS with isotropic one-dimensional analysis by electronic detector. For concentrations up to 10 wt%, a constant peak was observed, corresponding to 36 Å, due to the inner structure of the micelle, compatible with light scattering results, giving a hydrophobic radius of 16.7 Å (extended chain). 

The diversification included also a doctorate in binary amorphous alloys and more doctorates using several techniques. More students have been formed, and several papers have been published, but the focus here turns now to some specific problems that drew my attention over a long period, trying to join basic physical concepts with the behavior of these very complex systems. 

From the discovery of the nematic domain with SLS, studies went in the direction of a detailed exploration on the changes in the micelles at the phase sequence isotropic (I)–Hexagonal (H_α_) in the binary system SLS/water. The basic discussion was on the micellar radius, known to be that of the extended chain in spherical micelles in I phase. In the H_α_ phase, the distance between cylinders is given by X-ray diffraction, but the cylinder length is unknown, and it was usual to deduce the cylinder radius from the hypothesis of “infinite cylinders”, imposing homogeneous distribution of amphiphile and water in the plane perpendicular to the cylinders. This usually resulted in a cylinder paraffinic radius smaller than the extended length of the chain. The alternative was to admit that the cylinders are finite, with polydispersity in length, but keeping the same radius of the spherical micelle. 

This possibility was checked analyzing the variation of the hexagonal peaks with SLS concentration, compared with the cylindrical form factor calculated assuming a two-step radial distribution function (inner paraffinic region and an outer shell containing polar heads and hydration water) [42]. This means that the basic principle that matter density is the same at the atomic level and at the macroscopic level was extended to the transition I–H_α_. The analysis considered the relations between volumes occupied by micelles and water and some possible forms for the micelles, together with possibilities of both two-dimensional and three-dimensional short-range ordering. The results indicated [42] that in the concentrated I phase, micelles have stable dimensions and are slightly anisometric, with micellar growth at the I–H_α_ transition, up to 230 Å in the H_α_ phase.

From the new results with SLS, another basic question started to deserve further investigation, namely the direct Nc–Nd transition in the SLS system, without an intermediate biaxial phase [43]. 

The growth of micellar cylinders in SLS/water was further studied by analyzing the behavior of the hexagonal cell parameter ***a*** in the function of volume concentration ***c_v_*** along the phase sequence transitions I–H_α_–M_α_ (monoclinic distorted hexagonal). A functional behavior ***a*** ∝ ***c_v_***^−1/3^ was obtained [44], indicating tridimensional expansion, that is, finite length, instead of the coefficient −1/2 expected for only in-plane separation. 

At the same time, a new approach relating the micellar bending energy to the surfactant packing parameter for SLS indicated transformation of prolate ellipsoid into a spherocylinder for anisometry ν > 1.8 [45], a possible explanation for the transition I–H_α_. The subsequent question, related to the role of decanol in these lyotropic aggregates, is being investigated still, without a complete answer. This direction started with analysis of the sequence I–Nc–H_α_ in the ternary system SLS/water/decanol, after the determination of the nematic domain [41].

Instead of describing all the steps of the research done throughout more than three decades on this problem (including 3 Ph.D. these and many papers), it is more efficient to make reference to an invited paper published in the volume of Liquid Crystals in honor of Saupe, discussing the up-to-now unsolved problem of the micelles in the biaxial nematic phase [46], with 91 references.

However, the present state of the problem focusing on theoretical models is discussed in the next section.

In the context of this section, it is still worthwhile to mention that a detailed structural study of the H_α_ phase in SLS/water was performed, and the electron density maps obtained from the X-ray diffracted intensities allowed determination of the micellar parameters [47].

Regarding expertise in X-ray crystallography, the collaboration with the Italian group of Ancona began in the second decade with a further advance regarding the analysis of complex systems [48].

From the point of view of applications, lyotropic liquid crystals are now used as templates for a variety of nanostructures; in particular, the nematic Nc phase of SLS is used as templates for carbon nanotubes [49,50].

It is worthwhile also to mention that the nematic lyotropic system sodium dodecyl sulfate, decanol, and water was studied by rheology and deuteron nuclear magnetic resonance NMR measurements [51], showing strong evidence that the N_C_ and Nd nematics are textured nematics of the flow-aligning type, and not of the well-established tumbling-type surfactant nematics. 

### 4.5. The Nc-(Biaxial)-Nd Transition

When the existence of the two uniaxial nematic phases was discovered by chemists [32], they were classified as Type I and Type II, depending on their orientation on the magnetic field by NMR measurements on samples of detergent/deuterium/additives (decanol and /or salt), with positive and negative bulk diamagnetic anisotropy. A next paper was published [52] with a table summarizing all the phases known at that time, which already defined Type I as cylindrical (CM) and Type II as discotic (DM) micelles, and their relationship to the parent “hexagonal” and “lamellar” lyotropic liquid crystals. They also had different surface orientations, so that it was very clear that micelles changed symmetry with changes in composition. This was five months before the paper on the discovery of the biaxial phase [34], intermediate between the two uniaxial phases in a specific ternary system (with potassium laurate), investigated by temperature variation in a specific sample composition, related to the entrance of physicists into this field. 

Since the beginning, it was clear that there was a change in the symmetry of the micellar object, and that this could happen easily only in a lyotropic system, since thermotropic LC did not present a change in molecular symmetry. As already discussed in Section 4.4, and in the published review [46], the problem had no trivial solution. However, it is worthwhile to discuss in some detail the theoretical models that effectively approached experimental results.

A first attempt to model the symmetry transition, in collaboration with a theoretical Italian chemist continuing a work on the I–H_α_ transition [45], was an adaptation of the bending elastic theory of bilayers in vesicles to the micellar case, in terms of the surfactant parameter (already mentioned in item 4.3). The nematic cylindrical (Nc)-nematic discotic (Nd) phase transitions are correlated with a change of micellar form from spherocylinder (SC) to square tablet (ST) [53], which occurs geometrically in a continuous way, with an intermediate biaxial object. Good agreement was obtained for three amphiphile/decanol/water systems, where the transition occurs as a function of the decanol/amphiphile molecular ratio. 

This result inspired a Maier–Saupe model for a polydisperse solution of micelles of axial symmetry [54], giving a phase diagram with a biaxial phase at small average anisometries and finite dispersion in size. A further model [55] considered, with a statistical microscopic approach, a mixture of cylinder and disk micelles, with changes of uniaxial micellar form occurring either smoothly or abruptly, to mimic the possibilities of both the biaxial phase and coexistence. 

In another direction, Mukherjee in India [56] described new topologies in the phase diagrams involving biaxial nematic liquid crystals, finding a direct isotropic–biaxial nematic phase transition and three different biaxial nematic phases, outlining how the novel phase diagrams could be detected experimentally. 

Subsequent models with exact statistical mechanics calculations for a Maier–Saupe lattice model considered the inclusion of extra degrees of freedom to mimic a mixture of discs and cylinders. A quenched distribution of shapes leads to a phase diagram with two uniaxial and a biaxial nematic structure. A thermalized distribution, however, precludes the stability of this biaxial phase. The introduction of a two-temperature formalism (to mimic a separation of relaxation times) shows that a partial degree of annealing is already sufficient to stabilize a biaxial nematic structure [57,58]. 

Biaxiality could be the result of perpendicular alignment of uniaxial particles of cylinder-like and disc-like geometry in a mixture. A model for a general distribution of micellar anisometries could fit the original experimental data of Yu and Saupe’s well-known 1980 paper [34], yielding a bimodal distribution, with the presence of two quadrupoles referred to as objects of opposite symmetry [59], giving support to the rationalization of the biaxial phase for lyotropic systems in terms of a polydisperse mixture of rod-like and disc-like micelles.

Recent results by Mukherjee investigate the influence of an external magnetic field [60]. The possibility of the various phases, including the coexistence phases, is explored by means of variation of the concentration [61]. The model describes the first theoretical observation of the phase transition between two biaxial nematic phases. The theoretical predictions are found to be in good qualitative agreement with available experimental results.

Regarding experimental results, it is worth mentioning a very recent paper [62] with evidence of positive and negative biaxial phases, with a transition point determined by means of optical image processing, and a careful discussion of the available literature.

After the above discussion on lyotropic systems with symmetry transitions, it is also worthwhile to mention the possible thermotropic biaxial phase, which has been sought for already more than 40 years. Even before this, a biaxial phase was predicted theoretically, without mention of lyotropics.

Here, it is worthwhile to focus on some articles from this period.

First of all, a paper by Chadrasekhar [63] on optical studies carried out on a nematogenic copper complex, which incorporates the features of both rod-like and disk-like molecules, showing both a biaxial phase in the pure complex and a uniaxial–biaxial transition in binary mixtures with temperature variation.

In another interesting paper, measurements were carried out on two liquid crystalline organo-siloxane tetrapods [64], and results unambiguously showed the existence of a biaxial nematic phase below a uniaxial nematic phase.

A very recent paper considered colloidal particles suspended in liquid crystals [65], which exhibit various effective anisotropic interactions that can be tuned and utilized in self-assembly processes.

So many decades of investment clearly show that the solution is not a strict focus on “mathematical exactness” with no connection to experimental reality. 

## 5. Biological and Bio-Inspired Liquid Crystals

Biology is not an exact science. Studies on life have existed since antiquity in the ancestral practices of medicine, and Aristotle started systematization by comparison, classifying plants and animals, and separating animals those with blood and without blood. Only in the 17th century, based on careful observation of gardens since infancy, the Swedish scientist Carl Linnaeus proposed a more exact systematization in his work *Systema Naturae*, published in Latin in 1735, which is origin of the modern taxonomy. He used the word “biologie” in Latin, and the German translation of his book in 1771 also used “biologie”.

The invention of the microscope in Holland in the 17th century made possible the observation of bacteria and sperm, and with the advance of better microscopes, cells could be identified. Biology became a science at the turn of the 20th century, recognizing the cell as the basic unity of life, genes as the basic units of heredity, and evolution as the principle for changes in life.

Molecular biology starts with the determination of the helical structure of DNA, by X-ray diffraction, and attribution of nucleotides as its basic molecular structure. The history of the union of molecular biology with genetics and biochemistry can be followed in [66].

Molecular biology approaches exact science only when samples have an exact chemical composition, since only then can the analysis follow exact principles. Biological material does not in general satisfy such criteria. Academic work in biochemistry and biophysics is usually made on biomimetic systems, such that the effect of the components can be rationally studied in analytic form.

### 5.1. DNA and Lipids

Biology has always interested me in life. Its lack of exactness pushed me away during graduation, but attracted me afterwards. During the period of the reactor, I participated in studies of DNA samples by slow neutron transmission, at room temperature, comparing a dry sample and a wet sample with 7.8% moisture. The total neutron cross-section of the water was obtained, and the only conclusion was that it behaved as free water. After becoming the first woman Full Professor in IFUSP (1991), I was engaged in the creation of the Department of Applied Physics in IFUSP.

In the period of broader research directions (Section 4.2), a collaborative project with the laboratory at Ancona, Italy, started with LLC and continued in these systems of biological interest. A published article was written with the participation of a Brazilian student in Ancona on effect of a drug in a biomimetic membrane [67]. This was followed by a work with an Italian student in São Paulo [68], related to the same problem of the structure of the H_α_ phase, already discussed for SLS. This exchange of subjects of research lasted intermittently for almost 30 years, depending on mutual visits and interests.

A deoxyguanosine–water system, presenting an intermediate cholesteric Ch phase between isotropic I and hexagonal H_α_ phases, showed a behavior typical of infinite or long flexible cylinders in H_α_ phase, correlating the hexagonal parameter ***a*** with the lipid volume concentration ***c_v_***, according to ***a*** ∝ ***c_v_***^−1/2^ [68].

After this result, studies of micellar growth were extended from SLS to lipids, focusing on transitions to cubic lyotropic phases (Q), with measurements made in Europe [69,70]. There is much interest in the bicontinuous cubic phases with intricate structures, as sketched in Figure 3, both from the academic point of view and based on their possible existence in biological systems. Cubic phases with spherical micelles also exist between the I and H_α_ phases, which is also an intricate problem that drew my attention.

The lipid OLPC (oleoyl-lyso-phosphatidyl choline), presenting a cubic bicontinuous phase (Q^230^), has the phase sequence I–H_α_–Q–L_α_. The behavior measured in the H_α_ phase gave ***a*** ∝ ***c_v_****^−x^*, with *x* < 1/3 (in fact, *x* = 0.226), indicating that micelles grow from spherical in the I –H_α_ phase transition to infinite in the transition H_α_–Q^230^ [69]. It was possible to define a function representing the cylinder growth in the function of ***c_v_***. Results with SLS/water and SLS/water/decanol could also be compared with statistical mechanical calculations for systems with self-association. 

After that, three lipid–water systems (PLPC, OLPC, and DTAC) with transitions to cubic phases following the sequence I–H_α_–Q, either bicontinuous (Q^230^) or cubic micellar (Q^223^), received a more complete X-ray study [70]. PLPC (palmitoyl-lyso-phosphatidyl choline) has the phase sequence I–Q^223^–H_α_–L_α_, while DTAC (dodecyl–trimethyl-ammonium chloride) has the phase sequence I–Q^223^–H_α_–Q^230^–L_α_. The behavior of the hexagonal parameter and the electron density map were obtained, showing epitaxial relationships occurring at the H_α_–Q transitions, in the direction of the cylinder growth. It was possible to propose a mechanism for the transformation of the structures [70].

This type of study continued with another Ph.D. in our lab, focusing on the transition I–Q^223^ in PLPC from the isotropic side [71]. The more diluted region gives the particle form factor P(q), and in the more concentrated region, the SAXS curve is fitted by the product of P(q) S(q), with the structure factor calculated using a hard sphere interaction potential. It is found that PLPC micelles remain with a prolate ellipsoidal shape of constant anisometry ν = 1.80 ± 0.05 in the whole concentration range in the I phase. It is concluded that the formation of the Q^223^ phase requires that micelles remain spheroidal, in contrast with SLS, where micelles grow to slightly higher anisometry (ν∼2.4) before the I–H_α_ transition, in agreement with the elastic bending theory in terms of the surfactant parameter [45].

The collaboration with Italy extended to organic chemistry in Bologna, in a study with DNA carried out in our X-ray lab with samples from Italy [72]. Aqueous solutions of fragmented DNA were studied by SAXS, varying the concentration. The less concentrated solution (I phase) gives the DNA form factor, in good agreement with the B form of DNA. The semidilute regime until the I-cholesteric phase transition has an interference peak position that behaves with concentration with the exponent ½ for an effective rod length of L = 340 Å. The structure factor is obtained by dividing the SAXS curves by the form factor, and is modeled with Gaussian functions, with peak broadening varying also with the exponent ½, indicating a short-range order slightly above first neighbors.

The analysis of the short-range order of rodlike polyelectrolytes in terms of changes from exponent 1/3 to 1/2 when going from a dilute to semidilute regime (after a critical concentration) was extended in the following paper [73] to virus solutions taken from the literature. The number of ordered next neighbors arranged locally around a central particle indicates also only first neighbors, in good agreement with Monte Carlo results, when the ionic strength of the solution is low.

The transition I–Q^223^ in PLPC was further investigated in our laboratory, now from the side of the cubic micellar phase [74]. It was found that a local cubic order already exists in the concentrated I phase, but the positional correlation is between clusters with 5 micelles. In the lower concentration limit of the Q^223^ phase, the long-range order is established, but micelles have the same paraffinic volume as in the I phase.

### 5.2. A Special Biomembrane

**Figure 5:**
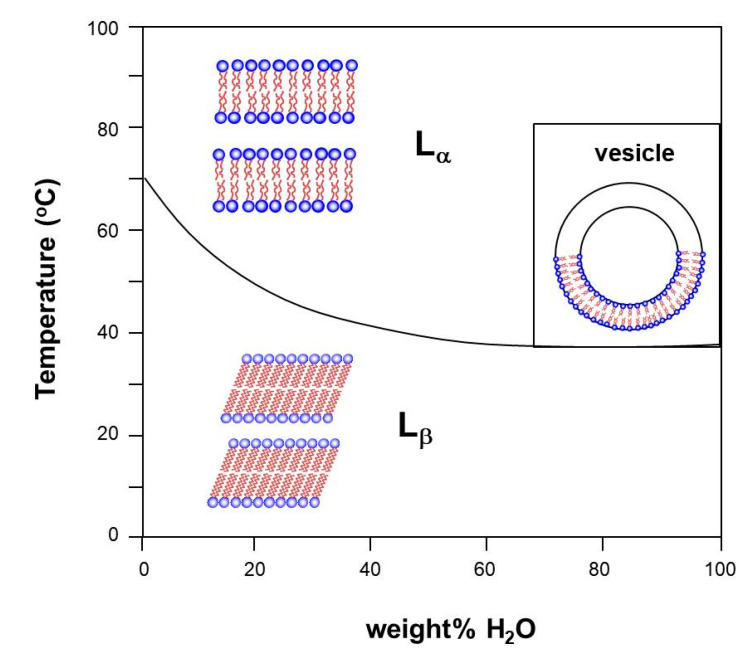
Phase diagram of usual phospholipid membranes, with order (Lamellar L_β_)–disorder (Lamellar L_α_) transition at the Krafft line, in the low-water-content region. Vesicles (unilamellar or multilamellar) are formed in the high-water-content region.

The next and final subject that drew my attention, with long-lasting research, was a special negatively charged phospholipid, with unusual phase transitions from the gel phase to the liquid crystalline phase. Figure 5 shows a scheme of the order–disorder phase transition of phospholipids in membranes, showing the separation between the high-concentration region, where LLCs are studied, and the low-concentration region, where vesicles are studied. 

Phosphatidylglycerol (DMPG, 1,2-dimyristoyl-sn-glycero-3-[phospho-rac-glycerol]) is the most abundant anionic phospholipid present in prokaryotic cell membranes and has been extensively studied as a model for negative membranes, in contrast to the neutral phosphatidylcholine (DMPC, 1,2-dimyristoyl-sn-glycero-3-[phosphocholine]). Figure 4 shows these phospholipid molecules, with two HC chains, compared with SLS, with one HC chain. 

DMPG was already being studied by the biophysics group at IFUSP at low concentrations, as usual (1 to 10 mM in HEPES buffer), by several techniques. The main transition showed, by light scattering, three different regions. At low (20 °C) and high temperatures (30 °C), the sample is turbid, whereas between the transitions, a transparent and viscous phase is observed. DMPG does not exist in eukaryotic cells, and my interest was in trying to correlate this absence with its unusual thermal behavior. 

I joined the team in structural studies using X-rays, which required higher concentrations (50 mM) for reasonable intensities. The initial focus on this subject resulted in a series of four articles in collaboration with the group at IFUSP, using the Brazilian synchrotron, with SAXS curves obtained with a linear position-sensitive detector [75,76,77,78]. 

The initial SAXS results [75] showed for DMPC the Bragg peak typical of multilamellar vesicles, with a repeat distance of 66 Å, while for DMPG, it was only a broad peak, indicating single bilayers, for the range of temperatures studied (10–45 °C). The peak position changed continuously in the intermediate region, while the intensity showed an unusual effect, with a decrease along the intermediate region and a minimum at its end, and bilayer electron density profiles fitted to the SAXS curves.

The light scattering thermal profile was similar to that yielded by the 10 mM DMPG dispersion, with only slight change in the temperature interval. In differential scanning calorimetry (DSC), DMPC presents a pretransition and a very sharp and strong melting peak at Tm = 23.5 °C. In contrast, a complex calorimetric profile is observed for 50 mM DMPG at low ionic strength, between 10 °C and 30 °C, besides the pretransition. At high ionic strength, this unusual thermal behavior disappears. The conclusion [75] was for a DMPG complex melting regime at low ionic strengths, between re-named temperatures T_m_^on^ and T_m_^off^, the onset and outset of the melting regime, respectively.

The next step was to decrease the minimum scattering angle measured in SAXS, in order to reach larger correlation distances. Besides the bilayer peak present in all phases, a peak corresponding to a mesoscopic structure at 400 Å was detected for DMPG only in the intermediate region [76]. The behavior of this repeat distance *d* was analyzed as a function of the lipid concentration ***c_v_***. For a bilayer, the symmetry decouples the three-dimensional volume partition of the lipid in a one-dimensional factor (perpendicular to the bilayer) and a two-dimensional factor (in the bilayer plane). For infinite planar bilayers with thickness *t*, ***c_v_*** coincides with the linear fraction of lipid occupancy (*t*/*d*). When the lamellae have fluctuations or are finite, it is possible to define a lipid surface fraction (LSF) such that ***c_v_*** ∝ LSF(*t*/*d*). The value LSF can be obtained from knowledge of ***c_v_***, *t*, and *d*, and it is <1, as shown in Figure 5 from [76], which is compatible with in-plane and not lamellar correlation.

In the same article, a new technique was used in the post doctorate of a Brazilian student in Germany, with observation of giant DMPG vesicles with phase contrast light microscopy, showing that vesicles ‘‘disappear’’ upon cooling below T_m_^off^ and ‘‘reappear’’ after reheating above T_m_^on^ [76]. It was proposed in this paper that the melting regime corresponds to unilamellar vesicles with perforations, with possible biological relevance.

The third paper in this series dealt with the effect of salt addition, with increase in ionic strength investigated by SAXS and optical microscopy (OM), indicating the changing of vesicles [77]. By SAXS, the broad bilayer peak arising from the electron density contrasts within the bilayer was observed at all temperatures up to 100 mM NaCl addition. At higher ionic strength (250–500 mM NaCl), an incipient lamellar repeat distance around d = 90–100 Å is detected, superimposed to the bilayer form factor, indicating a loose multi-lamellar order of only four bilayers, accounted for by DLVO theory [77].

The final paper in this series used DSC, turbidity, and OM of giant vesicles, combining phase and fluorescence microscopy to study the narrowing of the transition region with the increase of ionic strength [78]. It was possible to correlate the complex DSC profile with the vanishing of the bilayer optical contrast. The indication is that bilayers are perforated along the transition and the bilayer completely loses the optical contrast, directly related to turbidity.

The study of DMPG continued within the collaboration with the Ancona group, and my interest was in filling the gap in Figure 5 at intermediate concentrations. Another long-lasting series of three articles used new data at higher DMPG concentrations, obtained in the Brazilian synchrotron with SAXS and WAXS (wide angle for detection of melting of HC chains) and also POM [79,80,81]. The sodium salt of DMPG, and bidistilled water, were used to prepare samples with concentrations in the interval of 70–300 mM. The same buffer (10 mM HEPES pH 7.4 with 2 mM NaCl) was used throughout, without any further adjustment of pH of the lipid dispersion, to ensure the same additional low ionic strength as in all the previously published results on DMPG at smaller concentrations. However, the measured pH remained always above the apparent pK of DMPG, ensuring that DMPG can be assumed to be fully deprotonated and that the effects are due to changes in DMPG concentration. A thermal bath was used for temperature variation from 12 °C to 55 °C. 

At these higher concentrations, the melting regime persists, but it is not transparent. Defined SAXS peaks appear, and a new lamellar phase Lp with pores is proposed to exist above 70 mM DMPG, starting at 23 °C (3 °C above T_m_^on^) and losing correlation after T_m_^off^. A preliminary qualitative analysis of such a large amount of data led us to focus on SAXS curves with 150 mM DMPG to analyze the very complex behavior of the mesoscopic correlation peak with temperature [79]. The lipid surface fraction given in Figure 3 of [79] shows a near match of the previous results up to 70 mM in [76], and the new results up to 300 mM, providing evidence that Lp is not a normal bulk lamellar phase. In the sample with 150 mM, the DSC showed a clear peak corresponding to the new Lp phase, WAXS showed the continuous melting of the HC chains, and the intensity of the broad bilayer peak had a complex behavior. However, a pore model was developed and gave a good fit to the 70 mM curve in the intermediate region [79].

These data were analyzed in another paper, presented in an SAXS conference [80], using a model of water-penetrated bilayers (instead of pores) but without good agreement with the details of the variation of the intensity of the broad bilayer band.

Only six years later was the final paper published, with a detailed pore model fit to the experimental results [81]. Large and small toroidal pores are necessary to explain the SAXS results. Pores have DMPG in the fluid conformation, whereas the flat region of the bilayer has DMPG molecules in fluid and in gel conformations. Electron densities consider all molecular and ionic species that characterize the system and the temperature dependency of their volumes. The gel phase transforms initially, at 19.4 °C, in uncoupled bilayers with large pores, which transform into small pores along the lamellar phase. The minimum intensity of the SAXS bilayer peak at 30 °C corresponds to a maximum number of small pores, and above 35 °C, the system enters into the normal lamellar fluid phase, without pores. The charge is estimated and shows that the regions with pores contain fewer Na^+^ ions per polar head; hence, when they are forming, there is a release of Na^+^ ions toward the bulk.

The opening of pores by temperature is certainly of biological importance, and may explain why DMPG cannot exist in eukaryotic cells. With this final article, I finished my involvement with molecular biophysics, but my interest in biology continues.

### 5.3. Applications of Scientific Knowledge

This review has discussed, after an introduction, the relative importance of theory and experiment—a question which has no trivial answer, since knowledge is constructed from both. Now, the discussion is on the importance of applications of the acquired scientific knowledge, and some focus on the question of basic vs. applied science is in order. The search for knowledge in humans comes from innate curiosity, present from early childhood, and from the wish/necessity to understand the world around each one of us—not necessarily from material needs. Therefore, the drive for basic research is in general not the same as for applied research, and applications can develop only after basic knowledge has been acquired.

The next sections focused on my own trajectory, but inserted in a more general context of the study of condensed matter physics and soft matter, also approaching chemistry and biology. My aim is to link basic knowledge with innovative ideas.

I finish this section with some relevant interdisciplinary research taken from the literature, regarding interesting and novel applications in the direction of life. The comparison of basic properties of liposomes (spherical vesicle with lipid bilayers) and polymersomes (self-assembled block-copolymer vesicles) as cell-mimicking [82] allows a better a priori choice and design of vesicles. On the other hand, polymersomes have showed the ability to significantly enhance the efficacy of the antibiotics killing established intracellular pathogens and represent innovative applications for the eradication of intracellular bacteria [83].

A recent review on molecular bionics principles for supramolecular design [84] gives guidelines to design materials for biomedical applications, for tissue substitutes or in drug delivery vehicles, with applications in biomaterials engineering. 

## 6. Conclusions

This review did not mention temporary collaborations—for instance, works on microemulsions or cationic micelles—nor all of my academic research work. The focus has been on some non-trivial subjects which required my special attention, leading to series of correlated papers, pursuing solutions, and searching for internal consistency. Experimental and theoretical techniques are tools for research, but the real problem is the correct understanding of basic principles, unifying different branches of knowledge. 

My interest is in joining experimental results with simple theoretical approaches, which are able to illuminate and clarify unsolved and intriguing problems. A principle that emerged was the analysis of basic scaling laws’ impact on distribution of matter, correlating structures not completely defined in the three dimensions of space with accessible and known macroscopic molecular densities.

I could also mention efforts in the direction of teaching and scientific divulgation, in the field of these complex systems [85], and also work throughout my whole academic career applying physical methods to the study of human physical evolution [86].

## References

[1] Fultz B. (2020). Phase Transitions in Materials.

[2] Tilley R. (2020). Crystals and Crystal Structures.

[3] Menger F.M. (2002). Supramolecular chemistry and self-assembly. Proc. Natl. Acad. Sci. USA.

[4] Kralchevsky P., Miller R., Ravera F., Kralchevsky P., Miller R., Ravera F. (2019). Colloid and Interface Chemistry for Nanotechnology.

[5] (1995). Studies in Interface Science.

[6] Dunmur D. (2013). 125 years of liquid crystals. Liq. Cryst. Today.

[7] Gavroglu K., Simões A. (2012). Neither Physics nor Chemistry—A History of Quantum Chemistry.

[8] Bell E. (2001). The future of education in the molecular life sciences. Nat. Rev. Mol. Cell Biol..

[9] Jensen W.B. (2008). The Origin of the Polymer Concept. J. Chem. Educ..

[10] Gaitzsch J., Delahaye M., Poma A., Prez F.D., Battaglia G. (2016). Comparison of metal free polymer–dye conjugation strategies in protic solvents. Polym. Chem..

[11] Thorium in Brazil. http://www.aben.com.br/Arquivos/661/661.pdf.

[12] Breit G., Wigner E. (1936). Capture of Slow Neutrons. Phys. Rev..

[13] Fermi E. (1936). Motion of neutrons in hydrogenous substances. Ric. Sci..

[14] Zimmerman R.L., Amaral L.Q., Fulfaro R., Mattos M.C., Abreu M., Stasiulevicius R. (1967). Neutron Cross Section of Pr, Yb, Lu, Er, Ho, and Tm. Nucl. Phys. A.

[15] Van Hove L. (1954). Correlations in Space and Time and Born Approximation Scattering in Systems of Interacting Particles. Phys. Rev..

[16] Egelstaff P.A., Pease R.S. (1954). The design of cold neutron filters. J. Sci. Instrum..

[17] Larsson K.E., Bergstedt L. (1966). Proton Motions in Complex Hydrogenous Liquids. I. A Cross Section for Quasi-Elastic Scattering of Slow Neutrons. Phys. Rev..

[18] Larsson K.E., Amaral L.Q.D., Ivanchev N., Ripeanu S., Bergstedt L., Dahlborg U. (1966). Proton Motions in Complex Hydrogenous Liquids. II. Results Gained from Some Neutron-Scattering Experiments. Phys. Rev..

[19] Amaral L.Q., Vinhas L.A., Rodrigues C., Herdade S.B. (1968). Certain Aspects of the Calibration and Resolution of Slow Neutron Spectrometers. Nucl. Instrum. Methods.

[20] Allen H.C., Olson W.B. (1962). Vibrational-rotational spectroscopy. Annu. Rev. Phys. Chem..

[21] Krieger T.J., Nelkin M.S. (1957). Slow-Neutron Scattering by Molecules. Phys. Rev..

[22] Rush J.J., Taylor T.I., Havens W.W. (1961). Proton Motions in Solids by Slow Neutron Scattering Cross Sections. J. Chem. Phys..

[23] Herdade S.B. (1968). Slow neutron scattering and rotational freedom of methyl groups in several organic compounds. Proceedings of the Symposium on Neutron Inelastic Scattering.

[24] Rodrigues C., Vinhas L.A., Herdade S.B., Do Amaral L.Q. (1972). Slow-neutron scattering cross-section for methanol, ethanol, propanol, iso-propanol, butanol, ethanediol and propanetriol. J. Nucl. Energy.

[25] Rodrigues C., Amaral L.Q., Vinhas L.A., Herdade S.B. (1972). Proton Motion in Methanol by Cold Neutron Scattering. J. Chem. Phys..

[26] Amaral L.Q., Vinhas L.A., Herdade S.B. (1976). Methyl Rotation in Polydimethylsiloxane Studied by Neutron Transmission. J. Polym. Sci. Polym. Phys. Ed..

[27] Grapengeterl H.-H., Alefeld B., Kosfeld R. (1987). An investigation of micro-brownian motions in polydimethylsiloxane by complementary incoherent-neutron-scattering and nuclear-magnetic-resonance experiments below room temperature. Colloid Polym. Sci..

[28] Wolfa M.P., Salieb-Beugelaara G.B., Hunzikera P. (2018). PDMS with designer functionalities—Properties, modificationsstrategies, and applications. Prog. Polym. Sci..

[29] Amaral L.Q. (1972). Study of the Atomic Movements of t-Butanol by Slow Neutron Scattering. Ph.D. Thesis.

[30] Amaral L.Q., Fulfaro R., Vinhas L.A. (1975). Molecular Dynamics of Tert-Butanol Studied by Neutron Transmission. J. Chem. Phys..

[31] Amaral L.Q., Vinhas L.A. (1978). Molecular Dynamics of Tert-Butanol Studied by Neutron Inelastic Scattering. J. Chem. Phys..

[32] Radley K., Reeves L.W., Tracey A.S. (1976). Effect of Counterion Substitution on the Type and Nature of Nematic Lyotropic Phases from Nuclear Magnetic Resonance Studies. J. Phys. Chem..

[33] Amaral L.Q., Pimentel C.A., Tavares M.R., Vanin J.A. (1979). Study of a Magnetically Oriented Lyotropic Mesophase. J. Chem. Phys..

[34] Yu L.J., Saupe A. (1980). Observation of a Biaxial Nematic Phase in Potassium Laurate-1-Decanol-Water Mixtures. Phys. Rev. Lett..

[35] Amaral L.Q., Neto A.M.F. (1983). Interactions between Micelles in Nematic Lyomesophases. Mol. Cryst. Liq. Cryst..

[36] Amaral L.Q., Rossi W. (1983). Surface and Magnetic Orientation in a Type II Nematic Lyomesophase. Mol. Cryst. Liq. Cryst..

[37] Amaral L.Q. (1983). Magnetic Orientation of Nematic Lyomesophases. Mol. Cryst. Liq. Cryst..

[38] Amaral L.Q. (1984). The Lower Temperature Phase of a Nematic Lyomesophase System. J. Appl. Crystallogr..

[39] Amaral L.Q. (1985). Transitions in a Lyomesophase: A Study by Thermal Analysis and Electron Microscopy. Mol. Cryst. Liq. Cryst..

[40] Amaral L.Q., Helene M.E.M., Bittencourt D.R., Itri R. (1987). New nematic lyomesophase of sodium dodecyl sulfate. J. Phys. Chem..

[41] Amaral L.Q., Helene M.E.M. (1988). Nematic domain in the SLS/H_2_O/decanol system. J. Phys. Chem..

[42] Itri R., Amaral L.Q. (1990). Study of the isotropic—Hexagonal transition in the system SLS/H_2_O. J. Phys. Chem..

[43] Amaral L.Q. (1990). First-Order Transition between Nematic Phases in Lyotropic Liquid Crystals. Liq. Cryst..

[44] Amaral L.Q., Gulik A., Itri R., Mariani P. (1992). Micellar hexagonal phases in lyotropic liquid crystals. Phys. Rev. A.

[45] Taddei G., Amaral L.Q. (1992). Bending Energy and the relative stability of Micellar Forms. J. Phys. Chem..

[46] Amaral L.Q. (2010). Micelles forming biaxial lyotropic nematic phases. Liq. Cryst..

[47] Itri R., Amaral L.Q., Mariani P. (1996). Structure of the hexagonal phase of the sodium dodecyl sulfate and water system. Phys. Rev. E.

[48] Spinozzi F., Carsughi F., Mariani P., Teixeira C.V., Amaral L.Q. (2000). SAS from inhomogeneous particles with more than one domain of scattering density and arbitrary shape. J. Appl. Crystallogr..

[49] Lagerwall J.P.F., Scalia G., Haluska M., Dettlaff-Weglikowska U., Giesselmann F., Roth S. (2006). Simultaneous alignment and dispersion of carbon nanotubes with lyotropic liquid crystals. Phys. Stat. Sol. B.

[50] Lagerwall J.P.F., Scalia G., Haluska M., Dettlaff-Weglikowska U., Roth S., Giesselmann F. (2007). Nanotube alignment using lyotropic liquid crystals. Adv. Mater..

[51] Thiele T., Berreta J.-F., Muller S., Schmidt C. (2001). Rheology and nuclear magnetic resonance measurements under shear of sodium dodecyl sulfate/decanolwater nematics. J. Rheol..

[52] Forrest B.J., Reeves L.W. (1981). New Lyotropic Liquid Crystals Composed of Finite Nonsphericai Micelles. Chem. Rev..

[53] Amaral L.Q., Santin Filho O., Taddei G., Vila-Romeu N. (1997). Change in micelle form induced by cosurfactant addition in nematic lyotropic phases. Langmuir.

[54] Henriques E.F., Henriques V.B. (1997). Biaxial phases in polydisperse mean-field model solution of uniaxial micelles. J. Chem. Phys..

[55] Henriques E.F., Passos C.B., Henriques V.B., Amaral L.Q. (2008). Mixture of changing uniaxial micellar forms in lyotropic biaxial nematics. Liq. Cryst..

[56] Mukherjee P.K., Sen K. (2009). On a new topology in the phase diagram of biaxial nematic liquid crystals. J. Chem. Phys..

[57] Do Carmo E., Liarte D.B., Salinas S.R. (2010). Statistical models of mixtures with a biaxial nematic phase. Phys. Rev. E.

[58] Henriques E.F., Salinas S.R. (2012). Biaxial nematic phase in the Maier-Saupe model for a mixture of discs and cylinders. Eur. Phys. J. E.

[59] Henriques E.F., Henriques V.B., Krebs P.R. (2017). Bimodal form distribution from modelling biaxial lyotropic liquid crystal solutions through a polydisperse Maier–Saupe model. Liq. Cryst..

[60] Mukherjee P.K., Rahman M. (2013). Isotropic to biaxial nematic phase transition in an external magnetic field. Chem. Phys..

[61] Mukherjee P.K. (2016). New phase diagrams in the mixture of rods and plates of biaxial nematic liquid crystals. J. Mol. Liq..

[62] Santos O.R., Braga W.S., Luders D.D., Sampaio A.R., Kimura N.M., Simões M., Palangana A.J. (2021). Optical characterization of a biaxial nematic between uniaxial nematic lyotropic phases. Phase Transit..

[63] Chandrasekhar S., Ratna B.R., Sadashiva B.K., Raja V.N. (1988). A Thermotropic Biaxial Nematic Liquid Crystal. Mol. Cryst. Liq. Cryst..

[64] Merkel K., Kocot A., Vij J.K., Korlacki R., Mehl G.H., Meyer T. (2004). Thermotropic Biaxial Nematic Phase in Liquid Crystalline Organo-Siloxane Tetrapodes. Phys. Rev. Lett..

[65] Müller D., Alexander T.K., Kierfeld J. (2020). Chaining of hard disks in nematic needles: Particle-based simulation of colloidal interactions in liquid crystals. Sci. Rep..

[66] Morange M., Cobb M. (1998). A History of Molecular Biology.

[67] Colotto A., Mariani P., Ponzi-Bossi M.G., Rustichelli F., Albertini G., Amaral L.Q. (1992). Lipid-drug interaction: A structural analysis of pindolol effects on model membranes. Biochim. Biophys. Acta BBA Biomembr..

[68] Amaral L.Q., Itri R., Mariani P., Micheletto R. (1992). Structural study of the aggregates formed by the dinucleoside phosphate G2 in aqueous solution. Liq. Cryst..

[69] Mariani P., Amaral L.Q., Saturni L., Delacroix H. (1994). Hexagonal-Cubic Phase Transitions in Lipid Containing Systems: Epitaxial Relationships and Cylinder Growth. J. Phys. II.

[70] Mariani P., Amaral L.Q. (1994). Micellar Growth in Hexagonal Phases of Lipid Systems. Phys. Rev. E.

[71] Castelletto V., Itri R., Amaral L.Q. (1997). Micellar aggregates near the isotropic-cubic liquid crystal phase transition. J. Chem. Phys..

[72] Castelletto V., Itri R., Amaral L.Q., Spada G.P. (1995). Small-Angle X-Ray Scattering of DNA Fragments: Form and Interference Factors. Macromolecules.

[73] Castelletto V., Amaral L.Q. (1999). Short Range Order of Rodlike Polyelectrolytes in the Isotropic Phase. Macromolecules.

[74] Castelletto V., Amaral L.Q. (1999). Isotropic-Cubic Liquid Crystalline Phase Transition in Aqueous Solutions of PLPC. J. Phys. Chem. B.

[75] Riske K.A., Amaral L.Q., Lamy-Freund M.T. (2001). Thermal Transitions of DMPG bilayers in aqueous solution: SAXS structural studies. Biochim. Biophys. Acta.

[76] Riske K.A., Amaral L.Q., Döbereiner H.-G., Lamy M.T. (2004). Mesoscopic structure in the chain melting regime of anionic phospholipid vesicles: DMPG. Biophys. J..

[77] Fernandez R.M., Riske K.A., Amaral L.Q., Itri R., Lamy M.T. (2008). Influence of salt on the structure of DMPG studied by SAXS and optical microscopy. Biochim. Biophys. Acta BBA Biomembr..

[78] Riske K.A., Amaral L.Q., Lamy M.T. (2009). Extensive Bilayer Perforation Coupled with the Phase Transition Region of an Anionic Phospholipid. Langmuir.

[79] Spinozzi F., Paccamiccio L., Mariani P., Amaral L.Q. (2010). Melting Regime of the Anionic Phospholipid DMPG: New Lamellar Phase and Porous Bilayer Model. Langmuir.

[80] Spinozzi F., Mariani P., Paccamiccio L., Amaral L.Q. (2010). New lamellar phase with pores in the chain-melting regime of an anionic phospholipid dispersion. J. Phys. Conf. Ser..

[81] Spinozzi F., Amaral L.Q. (2016). Pore Model in the Melting Regime of a Lyotropic Biomembrane with an Anionic Phospholipid. Langmuir.

[82] Rideau E., Dimova R., Schwille P., Wurm F.R., Landfester K. (2018). Liposomes and polymersomes: A comparative review towards cell mimicking. Chem. Soc. Rev..

[83] Fenaroli F., Robertson J.D., Scarpa E., Gouveia V.M., Guglielmo C.D., De Pace C., Elks P.M., Poma A., Evangelopoulos D., Canseco J.O. (2020). Polymersomes Eradicating Intracellular Bacteria. ACS Nano.

[84] Rodríguez-Arco L., Poma A., Ruiz-Pérez L., Scarpa E., Ngamkham K., Battaglia G. (2019). Molecular bionics-engineering biomaterials at the molecular level using biological principles. Biomaterials.

[85] Amaral L.Q. (2014). Entre Sólidos e Líquidos: Uma Visão Contemporânea e Multidisciplinar Para O Ensino Medio.

[86] Amaral L.Q. (2008). Mechanical analysis of infant carrying in hominoids. Naturwissenschaften.

